# Population-based SEER analysis of survival in colorectal cancer patients with or without resection of lung and liver metastases

**DOI:** 10.1186/s12885-020-6710-1

**Published:** 2020-03-23

**Authors:** Alexander R. Siebenhüner, Ulrich Güller, Rene Warschkow

**Affiliations:** 1 Clinic for Medical Oncology and Hematology, University Hospital Zurich and University of Zurich, CH-8091 Zürich, Switzerland; 2 University Clinic for Visceral Surgery and Medicine, University Hospital Berne, CH-3010 Berne, Switzerland; 3 Onkologie und Hämatologiezentrum Stial STS AG, CH-3600 Thun, Switzerland; 4 Division of Medical Oncology and Hematology, Kantonsspital St. Gallen, CH-9007 St. Gallen, Switzerland; 5 Department of Surgery, Kantonsspital St. Gallen, CH-9007 St. Gallen, Switzerland; 6 Institute of Medical Biometry and Informatics, University Heidelberg, 69120 Heidelberg, Germany

**Keywords:** Colorectal cancer, Right and left sided tumor, Liver metastasis, Lung metastasis, Chemotherapy, Surgery, Surveillance epidemiology and end results database (SEER), Propensity score analysis

## Abstract

**Background:**

Approximately one third of all patients with CRC present with, or subsequently develop, colorectal liver metastases (CRLM). The objective of this population-based analysis was to assess the impact of resection of liver only, lung only and liver and lung metastases on survival in patients with metastatic colorectal cancer (mCRC) and resected primary tumor.

**Methods:**

Ten thousand three hundred twenty-five patients diagnosed with mCRC between 2010 and 2015 with resected primary were identified in the Surveillance, Epidemiology and End Results (SEER) database. Overall, (OS) and cancer-specific survival (CSS) were analyzed by Cox regression with multivariable, inverse propensity weight, near far matching and propensity score adjustment.

**Results:**

The majority (79.4%) of patients had only liver metastases, 7.8% only lung metastases and 12.8% metastases of lung and liver. 3-year OS was 44.5 and 27.5% for patients with and without metastasectomy (HR = 0.62, 95% CI: 0.58–0.65, *P* < 0.001). Metastasectomy uniformly improved CSS in patients with liver metastases (HR = 0.72, 95% CI: 0.67–0.77, *P* < 0.001) but not in patients with lung metastases (HR = 0.84, 95% CI: 0.62–1.12, *P* = 0.232) and combined liver and lung metastases (HR = 0.89, 95% CI: 0.75–1.06, *P* = 0.196) in multivariable analysis. Adjustment by inverse propensity weight, near far matching and propensity score and analysis of OS yielded similar results.

**Conclusions:**

This is the first SEER analysis assessing the impact of metastasectomy in mCRC patients with removed primary tumor on survival. The analysis provides compelling evidence of a statistically significant and clinically relevant increase in OS and CSS for liver resection but not for metastasectomy of lung or both sites.

## Background

Colorectal cancer (CRC) is one of the most frequent malignant tumors. Indeed, based on the latest update of the national cancer statistics at the United States, CRC has the third highest incidence in both men and in women [1]. The incidence rate is estimated to be 1.2 million per annum in the US, and more than 600,000 patients die from this cancer every year [2]. Approximately one third of all patients with CRC present with, or subsequently develop, colorectal liver metastases (CRLM). Moreover, another 23–38% of patients already have, or will develop extra-hepatic disease [3–5].

Over the past years the landscape of treatment modalities in patients with metastatic colorectal cancer has expanded tremendously and improved the median overall survival (OS) from a median of 5 months in 1993 [6] to more than 3 years nowadays [7]. Factors improving median survival rates of metastatic CRC are a better understanding of the heterogeneity of the disease based on rat sarcoma (RAS) and rapidly accelerated fibrosarcoma- (RAF) mutations as well as mismatch repair status, which allows a more patient-tailored treatment using antibody treatment in combination with chemotherapy or immunotherapy [8–12]. Moreover, the location of the primary cancer – left versus right hemicolon – has been recognized as important prognostic and predictive factor, particularly regarding the efficacy of epidermal growth factor receptor (EGFR) antibodies [13–16]. Despite this, rapidly accumulating knowledge about tumor heterogeneity of metastatic colorectal cancer (mCRC), many relevant questions regarding treatment sequences as well the impact and timing of resection of lung and liver metastases remain. The latest versions of national and international guidelines include resection of metastases at some point in the treatment of mCRC. However, these recommendations are often vague [17, 18]. While it is well recognized that liver resections in curative intent should be performed, there is ongoing debate regarding the resection of lung metastases and both lung and liver metastases.

Hence, the objective of the present population-based analysis was to assess the impact of resection of liver only, lung only and liver and lung metastases on survival in patients with mCRC with resected primary tumor.

## Methods

### Study cohort

The 2015 custom text data-version of the Surveillance, Epidemiology, and End Results (SEER) Program of the National Cancer Institute in the United States, covering approximately 28% of cancer cases in the United States, was the source of the present population-based analysis [19].

### Study design

**Fig. 1:**
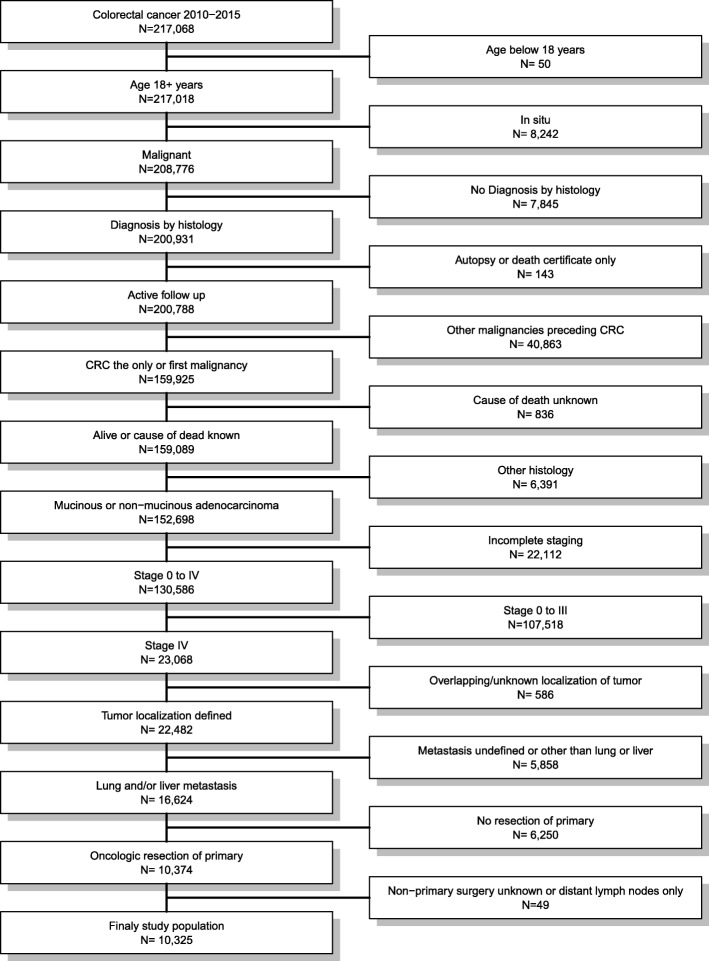
Flow chart of patients’ cohort definition. Data from the Surveillance, Epidemiology, and End Results (SEER) Program of the National Cancer Institute in the United States, covering approximately 28% of cancer cases in the United States were used for the present population-based analysis. Of 217,068 patients diagnosed with colorectal cancer between 2010 and 2015, 10,325 were eligible for analysis at the end of the selection process

All patients diagnosed with colorectal cancer between 2010 and 2015 were eligible for the analysis. Patients aged below 18 years, with missing diagnosis by histology, secondary malignancies preceding the mCRC, other histology than adenocarcinoma, incomplete staging, non-metastatic cancer, overlapping or unknown localization of the primary, metastasis other than liver and/or lung, undefined localization of the metastasis, not-resected primary, and unknown non − primary surgery or non − primary surgery limited to distant lymph nodes were excluded. Figure 1 depicts the selection process.

### Statistical methods

Statistical analyses were performed using the R statistical software (www.r-project.org). A two-sided *p*-value < 0.05 was considered statistically significant. Cancer-specific (CSS) and overall survivals were the co-primary endpoints. *P*-values were estimated using likelihood-ratio tests. The proportional hazard assumption for Cox regression was tested by scaled Schoenfeld residuals and by inspection of the hazard ratio (HR) plots [20]. After descriptive analysis, the imbalances regarding prognostic factors between patients with and without metastasectomy were assessed by multivariable logistic regression with adjustment for the site of the primary, metastatic site (lung, liver, both), T-stage, nodal status, grading, chemotherapy [21], year of diagnosis, age, gender, ethnicity, and marital status (risk set). The impact of metastasectomy on survival was analyzed with and without adjustment for the risk set. The impact of metastasectomy on survival was further analyzed with inverse propensity weight adjustment (stabilized weights) using the “ipw” R package [22]. Thereafter an exact matched propensity score and weighted analysis was performed as a superior and more refined statistical method for adjustment [23–25] using the “MatchIt” R package [26]. Each patient with metastasectomy was matched to all possible patients without with exactly the same values on all the covariates in the risk set, forming subclasses such that within each subclass both weighted groups have exactly the same covariate values. Patients without a counterpart among the patients in the other group were excluded from this analysis. Finally, a near-far matched analysis was performed to assess the impact of metastasectomy with adjustment for unobserved confounding variables. FIPS county codes were used as the instrumental variable to build an encouraged and discouraged group according to county codes with a high and low rate of metastasectomy. These two groups were then matched and analyzed in a paired Cox-regression model.

Thereafter, the entire analysis was repeated in each of the three groups according to the site of the metastasis using the same risk set except from the site of the metastasis.

## Results

### Patients characteristics

Ten thousand three hundred twenty-five of 217,068 patients diagnosed with colorectal cancer between 2010 and 2015 were included (Fig. 1). The median follow-up time was 15 months (interquartile range: 6 to 28 months) for all patients and 19 months (interquartile range: 8 to 35 months) for those alive at the end of follow up. At the end of follow-up, 4299 (41.6%) patients were alive, 5667 (54.9%) died from cancer and 359 (3.5%) from other reasons. Overall, 8195 (79.4%) patients had liver metastases only, 807 (7.8%) patients presented with lung metastases only and 1323 (12.8%) presented with metastases on both sites (Table 1). Metastasectomy was performed in 2906 (28.1%) patients. The multivariable logistic regression confirmed the significant imbalances in the baseline characteristics between patients with and without metastasectomy for the entire risk set except for the site of the primary, T-stage and ethnicity.

**Table 1:** Patient characteristics

		Total *N* = 10,325	Resection			Metastasis			
			No Resection *N* = 7419	Resection *N* = 2906	P^a^	Liver *N* = 8195	Lung *N* = 807	Both *N* = 1323	P^a^
Resection	No	7419 (71.9%)	7419 (100%)	–	–	5695 (69.5%)	663 (82.2%)	1061 (80.2%)	< 0.001
	Yes	2906 (28.1%)	–	2906 (100%)		2500 (30.5%)	144 (17.8%)	262 (19.8%)	
Metastasis	Liver only	8195 (79.4%)	5695 (76.8%)	2500 (86.0%)	< 0.001	8195 (100%)	–	–	–
	Lung only	807 (7.8%)	663 (8.9%)	144 (5.0%)		–	807 (100%)	–	
	Liver and Lung	1323 (12.8%)	1061 (14.3%)	262 (9.0%)		–	–	1323 (100%)	
Tumor localization	Right colon	3485 (33.8%)	2602 (35.1%)	883 (30.4%)	< 0.001	2808 (34.3%)	220 (27.3%)	457 (34.5%)	< 0.001
	Transverse	1401 (13.6%)	1005 (13.5%)	396 (13.6%)		1140 (13.9%)	98 (12.1%)	163 (12.3%)	
	Left colon	3311 (32.1%)	2346 (31.6%)	965 (33.2%)		2666 (32.5%)	231 (28.6%)	414 (31.3%)	
	Rectosigmoid	966 (9.4%)	680 (9.2%)	286 (9.8%)		736 (9.0%)	88 (10.9%)	142 (10.7%)	
	Rectum	1162 (11.3%)	786 (10.6%)	376 (12.9%)		845 (10.3%)	170 (21.1%)	147 (11.1%)	
T-Stage	T1 to T3	6643 (64.3%)	4727 (63.7%)	1916 (65.9%)	0.034	5348 (65.3%)	532 (65.9%)	763 (57.7%)	< 0.001
	T4	3682 (35.7%)	2692 (36.3%)	990 (34.1%)		2847 (34.7%)	275 (34.1%)	560 (42.3%)	
N-Stage	N0	2103 (20.4%)	1439 (19.4%)	664 (22.8%)	< 0.001	1654 (20.2%)	230 (28.5%)	219 (16.6%)	< 0.001
	N+	8222 (79.6%)	5980 (80.6%)	2242 (77.2%)		6541 (79.8%)	577 (71.5%)	1104 (83.4%)	
Grade	G1/2	7715 (74.7%)	5450 (73.5%)	2265 (77.9%)	< 0.001	6090 (74.3%)	615 (76.2%)	1010 (76.3%)	0.118
	G3/4	2610 (25.3%)	1969 (26.5%)	641 (22.1%)		2105 (25.7%)	192 (23.8%)	313 (23.7%)	
Chemo- and/or	No	2842 (27.5%)	2294 (30.9%)	548 (18.9%)	< 0.001	2216 (27.0%)	235 (29.1%)	391 (29.6%)	0.437
Radiotherapy	Yes	7483 (72.5%)	5125 (69.1%)	2358 (81.1%)		5979 (73.0%)	572 (70.9%)	932 (70.4%)	
Year of diagnosis	2010–2012	5304 (51.4%)	3915 (52.8%)	1389 (47.8%)	< 0.001	4279 (52.2%)	365 (45.2%)	660 (49.9%)	0.005
	2013–2015	5021 (48.6%)	3504 (47.2%)	1517 (52.2%)		3916 (47.8%)	442 (54.8%)	663 (50.1%)	
Age (years)	< 50	1799 (17.4%)	1130 (15.2%)	669 (23.0%)	< 0.001	1474 (18.0%)	120 (14.9%)	205 (15.5%)	0.005
	50–64	4065 (39.4%)	2843 (38.3%)	1222 (42.1%)		3241 (39.5%)	285 (35.3%)	539 (40.7%)	
	65–79	3247 (31.4%)	2448 (33.0%)	799 (27.5%)		2525 (30.8%)	285 (35.3%)	437 (33.0%)	
	80+	1214 (11.8%)	998 (13.5%)	216 (7.4%)		955 (11.7%)	117 (14.5%)	142 (10.7%)	
Gender	Male	5693 (55.1%)	4159 (56.1%)	1534 (52.8%)	0.003	4585 (55.9%)	387 (48.0%)	721 (54.5%)	0.003
	Female	4632 (44.9%)	3260 (43.9%)	1372 (47.2%)		3610 (44.1%)	420 (52.0%)	602 (45.5%)	
Ethnicity	Caucasian	7787 (75.4%)	5576 (75.2%)	2211 (76.1%)	0.336	6237 (76.1%)	591 (73.2%)	959 (72.5%)	0.117
	African-American	1555 (15.1%)	1117 (15.1%)	438 (15.1%)		1219 (14.9%)	115 (14.3%)	221 (16.7%)	
	Other/Unknown	983 (9.5%)	726 (9.8%)	257 (8.8%)		739 (9.0%)	101 (12.5%)	143 (10.8%)	
Marital status	Married	5583 (54.1%)	3925 (52.9%)	1658 (57.1%)	< 0.001	4501 (54.9%)	399 (49.4%)	683 (51.6%)	0.061
	Single/Widowed	3057 (29.6%)	2256 (30.4%)	801 (27.6%)		2398 (29.3%)	259 (32.1%)	400 (30.2%)	
	Other/Unknown	1685 (16.3%)	1238 (16.7%)	447 (15.4%)		1296 (15.8%)	149 (18.5%)	240 (18.1%)	

n (percent)

^a^Chi-squared test

### Impact of metastasectomy on survival for all metastatic sites

The median CSS in patients with and without metastasectomy was 2.8 and 1.8 years and the 3-year survival rates were 46.3% (95% confidence interval (CI): 44.1–48.7%) and 29.4% (95% CI: 28.1–30.7%, HR = 0.62, 95% CI: 0.58–0.66, *P* < 0.001), respectively. The median OS in patients with and without metastasectomy was 2.6 and 1.7 years and the 3-year survival rates were 44.5% (95% CI: 42.3–46.8%) and 27.5% (95% CI: 26.2–28.7%, HR = 0.62, 95% CI: 0.58–0.65, *P* < 0.001), respectively. In multivariable analysis, metastasectomy was associated with improved CSS (HR = 0.75, 95% CI: 0.70–0.80, *P* < 0.001) and OS (HR = 0.75, 95% CI: 0.70–0.80, *P* < 0.001). Survival was better when the metastatic site was in the lung only and worse when occurring on both liver and lung data (Table 2). Survival was better in left sided colonic and rectal cancer, in nodal negative patients with lower T-stage and lower graded tumors and in younger, married, Caucasian patients who underwent chemotherapy (Table 2).

**Table 2:** Prognostic factors for overall and cancer-specific survival

		overall survival using Cox regression				cancer-specific survival using Cox regression			
		unadjusted^a^		full model^b^		unadjusted^a^		full model^b^	
		HR (95% CI)	p^c^	HR (95% CI)	p^c^	HR (95% CI)	p^c^	HR (95% CI)	p^c^
Resection	No	Reference	< 0.001	Reference	< 0.001	Reference	< 0.001	Reference	< 0.001
	Yes	0.62 (0.58–0.65)		0.75 (0.70–0.80)		0.62 (0.58–0.66)		0.75 (0.70–0.80)	
Metastasis	Liver only	Reference	< 0.001	Reference	< 0.001	Reference	< 0.001	Reference	< 0.001
	Lung only	0.78 (0.70–0.86)		0.69 (0.62–0.76)		0.75 (0.68–0.84)		0.67 (0.60–0.75)	
	Liver and Lung	1.62 (1.51–1.74)		1.58 (1.47–1.69)		1.64 (1.52–1.76)		1.59 (1.48–1.71)	
Tumor localization	Right	Reference	< 0.001	Reference	< 0.001	Reference	< 0.001	Reference	< 0.001
	Transverse	0.91 (0.84–0.98)		1.01 (0.93–1.09)		0.91 (0.84–0.98)		1.01 (0.93–1.10)	
	Left	0.61 (0.57–0.65)		0.77 (0.72–0.82)		0.61 (0.57–0.65)		0.77 (0.72–0.82)	
	Rectosigmoid	0.58 (0.53–0.64)		0.72 (0.66–0.80)		0.58 (0.53–0.64)		0.73 (0.66–0.80)	
	Rectum	0.46 (0.42–0.50)		0.75 (0.68–0.83)		0.46 (0.41–0.50)		0.75 (0.68–0.83)	
T-Stage	T1 to T3	Reference	< 0.001	Reference	< 0.001	Reference	< 0.001	Reference	< 0.001
	T4	1.60 (1.52–1.69)		1.44 (1.37–1.52)		1.74 (1.62–1.87)		1.47 (1.40–1.56)	
N-Stage	N0	Reference	< 0.001	Reference	< 0.001	Reference	< 0.001	Reference	< 0.001
	N1	1.64 (1.54–1.76)		1.57 (1.47–1.69)		1.74 (1.62–1.87)		1.65 (1.53–1.78)	
Grade	G1/2	Reference	< 0.001	Reference	< 0.001	Reference	< 0.001	Reference	< 0.001
	G3/4	1.73 (1.63–1.82)		1.50 (1.42–1.59)		1.77 (1.67–1.87)		1.53 (1.44–1.62)	
Chemo- and/or	No	Reference	< 0.001	Reference	< 0.001	Reference	< 0.001	Reference	< 0.001
Radiotherapy	Yes	0.29 (0.27–0.30)		0.34 (0.32–0.36)		0.30 (0.28–0.31)		0.35 (0.33–0.37)	
Year of diagnosis	2010–2012	Reference	< 0.001	Reference	0.044	Reference	< 0.001	Reference	0.052
	2013–2015	0.89 (0.84–0.94)		0.94 (0.89–1.00)		0.89 (0.84–0.94)		0.94 (0.89–1.00)	
Age (years)	< 50	Reference	< 0.001	Reference	< 0.001	Reference	< 0.001	Reference	< 0.001
	50–64	1.24 (1.14–1.34)		1.12 (1.03–1.21)		1.21 (1.12–1.31)		1.10 (1.01–1.19)	
	65–79	1.71 (1.58–1.85)		1.35 (1.25–1.47)		1.62 (1.50–1.76)		1.29 (1.19–1.41)	
	80+	3.59 (3.28–3.94)		1.96 (1.77–2.17)		3.35 (3.05–3.69)		1.85 (1.67–2.05)	
Gender	male	Reference	0.106	Reference	0.048	Reference	0.034	Reference	0.2227
	female	1.04 (0.99–1.10)		0.95 (0.90–1.00)		1.06 (1.00–1.12)		0.97 (0.92–1.02)	
Ethnicity	Caucasian	Reference	< 0.001	Reference	< 0.001	Reference	< 0.001	Reference	0.001
	African-American	1.15 (1.08–1.24)		1.11 (1.04–1.20)		1.15 (1.08–1.24)		1.11 (1.03–1.20)	
	other/unknown	0.91 (0.83–1.00)		0.91 (0.83–0.99)		0.90 (0.82–0.99)		0.89 (0.81–0.98)	
Marital status	married	Reference	< 0.001	Reference	< 0.001	Reference	< 0.001	Reference	< 0.001
	single	1.46 (1.38–1.55)		1.21 (1.14–1.28)		1.45 (1.36–1.53)		1.20 (1.13–1.28)	
	other/unknown	1.12 (1.05–1.21)		1.05 (0.98–1.13)		1.09 (1.01–1.17)		1.02 (0.94–1.10)	

Hazard ratios (HR) with 95% confidence intervals (CI) of Wald type

^a^univariate Cox regression analysis; ^b^multivariable Cox regression analysis; ^c^likelihood ratio test

### Impact of metastasectomy on survival stratified for the metastatic sites

Metastasectomy was performed in 2500 of 8195 (30.5%) patients with exclusively liver metastases, in 144 of 807 (17.8%) patients with exclusively lung metastases and in 262 of 1323 (19.8%) patients with metastases on both sites. Figure 3 summarizes the impact of metastasectomy for OS and CSS in stratified analyses performed separately for the three metastatic site groups.

### Resection of liver metastases only

**Fig. 2:**
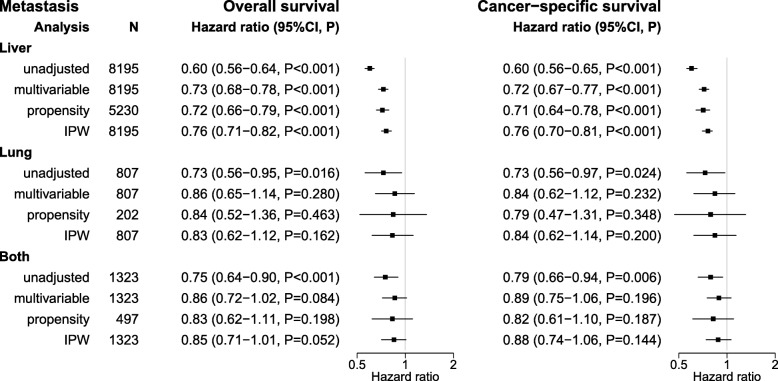
Forest plot of unadjusted and adjusted survival analysis stratified for metastatic site Analysis of survival was performed separately for patients with liver only, lung only, lung and liver being the metastatic site. The 95% confidence intervals for the hazard ratios were estimated using the Wald method and the *P*-values using the likelihood ratio test. The figure depicts the results for OS and CSS in unadjusted, multivariable adjusted, inverse propensity weight (IPW)-adjusted and exact matching and weighting propensity score adjusted analysis.

**Fig. 3:**
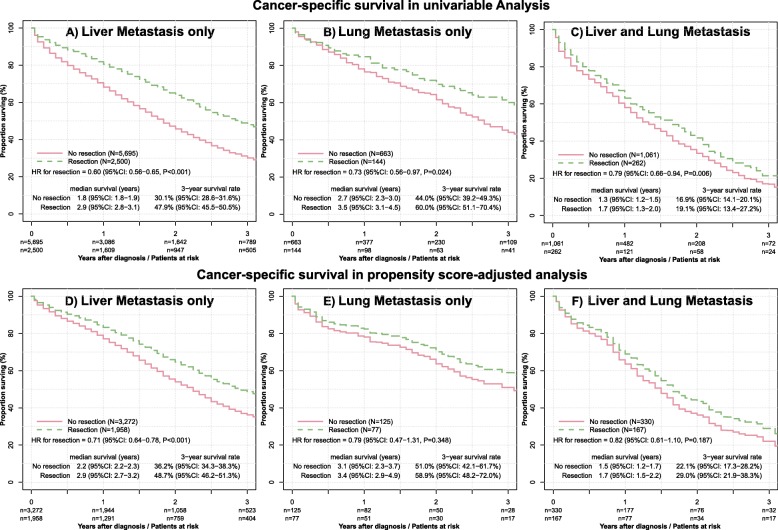
Unadjusted and PS-adjusted survival analysis stratified for metastatic site. The upper three plots display the survival curves for cancer-specific survival in unadjusted analysis for patients with liver, lung and both metastasis with and without resection (Panel A to C). The lower three plots display the survival curves after exact propensity matching (Panel D to F)

The stratified analysis demonstrated liver being the only metastatic site, for which metastasectomy was uniformly beneficial regarding OS and CSS in unadjusted and all adjusted analyses (Figs. 2 and 3).

### Resection of lung metastases only

For lung as the only metastatic site, metastasectomy was beneficial only in unadjusted analysis. The median OS in patients with and without lung metastasectomy was 3.3 and 2.5 years and the 3-year survival rates were 58.0% (95% CI: 49.1–68.4%) and 40.9% (95% CI: 36.3–46.1%) (HR = 0.73, 95% CI: 0.56–0.95, *P* = 0.016). However, after multivariable adjustment, lung metastasectomy did not have a significant impact on OS (HR = 0.86, 95% CI: 0.65–1.14, *P* = 0.280) and CSS (HR = 0.84, 95% CI: 0.62–1.12, *P* = 0.232). Furthermore, no benefit on CSS and OS was found in patients undergoing resection of lung metastases after propensity score- (PS) and inverse probability weighted- (IPW) adjustment (Figs. 2 and 3).

### Resection of combined lung and liver metastases

In patients with both lung and liver metastases, metastasectomy was beneficial only in unadjusted analysis. The median overall survivals in patients with and without metastasectomy were 1.5 and 1.2 years. However, none of the adjusted analyses yielded a survival benefit for patients undergoing metastasectomy (Figs. 2 and 3).

## Discussion

This is the first SEER analysis using IPW and PS to assess the impact of metastasectomy on survival in colorectal cancer patients with special focus on liver and/or lung metastases and with removed primary tumor. The present analysis provides compelling evidence of a statistically significant and clinically relevant increase in OS and CSS for liver resection but not for metastasectomy of lung or both sites.

Outcomes of patients with metastatic colorectal cancer (mCRC) have improved enormously over the last decade. Indeed, depending on the extent of metastases and the biology, the median OS of metastatic colorectal cancer patients can exceed 3 years [27]. There are different factors, which led to improved outcomes in patients with metastatic colorectal cancer: first, our knowledge of the tumor heterogeneity based on molecular profiling has changed the therapeutic management of these tumors. Thus, the more individual systemic treatment results in higher response rates and consecutively higher rates of surgical metastasectomy. These therapeutic concepts are well approved in large randomized trials in the first line as well second line settings [11, 28]. As refractory patients will be seen more frequently in sequential treatment of mCRC re-challenge concepts have been investigated with promising results [29, 30].

However, the therapeutic concepts of mCRC do not only contain chemotherapy or antibodies. Resections of oligometastatic liver and or lung metastasis are commonly discussed during multidisciplinary tumor boards and up to 15% of mCRC patients are evaluated for resection. The surgical management along the current guidelines of National Comprehensive Cancer Network (NCCN) and European Society for Medical Oncology (ESMO) is not restricted to one single organ as well no clear definition on the number of metastasis at liver or lung will restrict such procedures within a multidisciplinary approach to mCRC patients [17, 18]. However, good prognostic and predictive makers to guide this decision are still missing.

The benefit of metastatic resection for these selected patients led to an improvement of 5-years survival of 20–38% [31–33]. Most available data supporting resection of liver and or lung metastases are based on small case series as well retrospective data and reviews [34, 35]. Large cohort analyses to address the impact of metastasectomy in relation to OS or CSS are scarse.

To our knowledge this is the first population-based, propensity score adjusted analysis investigating the prognostic impact of resection of liver and or lung metastases in mCRC patients. Being aware of the conflicting data and the challenge to handle relevant bias due to substantial imbalances between resected versus not resected mCRC patients, we have intentionally selected the propensity score matching as a superior and refined statistical method in addition to commonly used multivariate analysis.

Based on a large collective from the SEER database of patients with metastatic colorectal cancer between 2010 and 2015 with resected primary tumor, the present study provides compelling evidence that the prognosis of patients with resection of liver metastasis in the overall population is better after adjusting for a strong bias regarding various patient and tumor characteristics by the use of the propensity score matching. Conversely, no differences in OS and CSS appeared in the propensity score adjusted population for the patients undergoing resection of lung metastases or both lung and liver metastases. Thus, we conclude that the overall survival improvement in mCRC patients after resection of lung and both lung and liver metastases described in the scientific literature are not real on a population-based level but caused by differences regarding confounders that could not be adjusted for in multivariate regression analysis.

Our results differ from the finding from Boysen et al. [36] demonstrating a survival benefit of lung metastasectomy compared to the group of no resection. However, in this Danish cohort study the benefit for lung metastasectomy was only found in univariate analyses and non-significant benefit was seen at their multivariate analysis for lung metastasectomy, hence selection bias (healthier patients with less metastatic disease, and better biology get operated) is inherent. Luo et colleagues [37] demonstrated in their SEER database analysis that the metastatic site of patients with mCRC has prognostic impact. Indeed, isolated liver metastases have a better prognosis compared to metastases at multiple sites. However, the authors did not analyze the impact on outcomes of resection and survival of mCRC patients.

In highly selected patients there might be a benefit for a sequential metastasectomy of liver followed by systemic treatment followed by metastasectomy of the lung. However, the patient numbers in published analyses are low and without control group [38–42]. The same trend of survival benefit was reported for patients with mCRC after lung resection [43–45]. The limitations of these studies are the retrospective nature, inclusion of a highly selective patient cohort and most of them were done at a highly specialized cancer center and hence lack the generalizability to other hospitals. Most importantly, no thorough risk-adjustment was performed with propensity-score analyses and hence relevant selection bias is an inherent shortcoming. This explains the different results compared to our study, in which we aimed to properly address and limit selection bias.

Our study has some limitations, most of them by the lack of information which were not available from the SEER database. In fact, the SEER database does not provide any information about the intention or the extend of metastasectomy. Hence, we can not ascertain if all the three groups underwent metastasectomy with curative intention. However, there is some evidence that resection of lung metastasis in the setting of liver and lung metastasis in mCRC does not improve the survival [46], which is in accordance to our findings.

One limitation is the lack of biomarker information regarding RAS and rapidly accelerated fibrosarcoma isoform B (BRAF) mutations as well as microsatellite instability. Second, no information concerning the choice of systemic therapy as well the status of conversion rate for resection of initially unresectable metastasis are available. Third, information regarding the time-point of resection, type of surgery as well the resection margin (R0 or R1) was lacking. Fourth, the SEER database does not provide any information regarding the diagnosis of pulmonary metastasis. Hence it is possible, that small pulmonary nodules in computed tomography (CT) scans were benign. However, due to the population-based nature of this analysis that reflects the real United States population with metastatic colon cancer, the lack of this information does not impact our results, albeit limits the extent of interpretation of our data.

## Conclusion

This population-based propensity score adjusted analysis of mCRC patients with liver and or lung metastases provides compelling evidence that the resection of liver metastases improves OS and CSS. In contrary, the resection lung metastases as well as both lung and liver metastases did not result in increased survival.

## Data Availability

The data used in this study are available free of charge online at www.seer.cancer.gov on request. All data generated or analyzed during this study are included in this published article.

## Abbreviations

BRAF: Rapidly accelerated fibrosarcoma isoform B
CI: Confidence interval
CRC: Colorectal cancer
CRLM: Colorectal liver metastases
CSS: Cancer specific survival
CT: Computed tomography
EGFR: Epidermal growth factor
ESMO: European Society for Medical Network
HR: Hazard ratio
IPW: Inverse probability weighted
mCRC: metastatic colorectal cancer
NCCN: National Comprehensive Cancer Network
OS: Overall survival
PS: Propensity score
RAF: Rapidly accelerated fibrosarcoma
RAS: Rat sarcoma
SEER: Surveillance epidemiology and end results database
